# Can Shockwave Treatment Elicit a Molecular Response to Enhance Clinical Outcomes in Pressure Ulcers? The SHOck Waves in wouNds Project

**DOI:** 10.3390/biomedicines12020359

**Published:** 2024-02-03

**Authors:** Mirosław Sopel, Izabela Kuberka, Izabela Szczuka, Jakub Taradaj, Joanna Rosińczuk, Robert Dymarek

**Affiliations:** 1 Department of Preclinical Sciences, Pharmacology and Medical Diagnostics, Faculty of Medicine, Wrocław University of Science and Technology, 50-370 Wroclaw, Poland; mirek.sopel@gmail.com; 2 Department of Anaesthetic and Surgical Nursing, Faculty of Health Sciences, Wroclaw Medical University, 51-618 Wroclaw, Poland; izabela.kuberka@umw.edu.pl; 3 Laboratory of Cells Propagation and Modification, Lower Silesian Oncology Hematology and Pulmonology Center, 53-413 Wroclaw, Poland; driszczuka@gmail.com; 4 Institute of Physiotherapy and Health Sciences, Academy of Physical Education in Katowice, 40-065 Katowice, Poland; j.taradaj@awf.katowice.pl; 5 Department of Internal Medicine Nursing, Faculty of Health Sciences, Wroclaw Medical University, 51-618 Wroclaw, Poland; joanna.rosinczuk@umw.edu.pl; 6 Department of Physiotherapy, Faculty of Health Sciences, Wroclaw Medical University, 50-368 Wroclaw, Poland

**Keywords:** extracorporeal shock wave, soft tissue injury, chronic wounds, pressure ulcers, histomorphology, immunocytochemistry, proliferation index, micro-vessels’ density, myofibroblasts, clinical outcomes

## Abstract

Wound healing requires the coordinated interaction of dermis cells, the proper deposition of extracellular matrix, re-epithelialization, and angiogenesis. Extracorporeal shock wave (ESW) is a promising therapeutic modality for chronic wounds. This study determined the biological mechanisms activated under ESW, facilitating the healing of pressure ulcers (PUs). A group of 10 patients with PUs received two sessions of radial ESW (300 + 100 pulses, 2.5 bars, 0.15 mJ/mm^2^, 5 Hz). Histomorphological and immunocytochemical assessments were performed on tissue sections obtained from the wound edges before the ESW (M0) and after the first (M1) and second (M2) ESW. The proliferation index of keratinocytes and fibroblasts (Ki-67), the micro-vessels’ density (CD31), and the number of myofibroblasts (α-SMA) were evaluated. The involvement of the yes-associated protein (YAP1) in sensing mechanical strain, and whether the nuclear localization of YAP1, was shown. The increased proliferative activity of epidermal cells and skin fibroblasts and the increased number of myofibroblasts, often visible as integrated cell bands, were also demonstrated as an effect of wound exposure to an ESW. The results indicate that the major skin cells, keratinocytes, and fibroblasts are mechanosensitive. They intensify proliferation and extracellular matrix remodeling in response to mechanical stress. A significant improvement in clinical wound parameters was also observed.

## 1. Introduction

The skin is the largest human organ in terms of its area and volume. It protects internal tissues against mechanical damage, infections, ultraviolet radiation, and extreme temperature. Unfortunately, the functions of the skin make it very susceptible to injuries, which is important not only for the patients themselves but also for the economy of healthcare [1,2]. Patients with pressure ulcers (PUs), especially the elderly, are predisposed to abnormal wound healing and its chronic treatment [3]. Wound healing requires the coordinated interaction of the dermis cells, the proper deposition of the extracellular matrix, re-epithelialization, and an angiogenic response. Increased cell proliferation is a prerequisite for producing new tissue to complement the defects caused by the injury [4].

Despite the many physical modalities in wound management such as electrotherapy, sonotherapy, and laser therapy, most of them are moderately effective and still require reliable verification. Therefore, there is a need to introduce more effective and scientifically better-verified wound healing methods into therapy [5,6]. Extracorporeal shock waves (ESWs) are a promising therapeutic agent for chronic wounds [7]. The advantages of ESWs compared to other therapeutic interventions include their non-invasiveness, low risk of complications, and cost-effectiveness [8].

Several animal models and in vitro studies proposed the action mechanisms of focused ESWs in clinical settings. For instance, in vitro exposure of cells to ESWs can influence their proliferation, differentiation, gene expression, production of growth factors, and release of cytokines [9,10,11,12]. Other studies have hypothesized that ESW sessions can induce molecular changes through mechanotransduction [5,12,13,14]. However, the molecular and cellular action mechanisms of ESWs are unknown to a large extent.

This study emphasized the importance of biological mechanisms activated under ESW interventions, facilitating the healing of PUs. The analysis included the proliferation index of keratinocytes and fibroblasts (Ki-67 expression), microvascular density (evaluation of CD 31 antigen expression), and the number of myofibroblasts (α-SMA expression). In addition, the study investigated whether the transcriptional cofactor YAP1 was involved in the sensing of mechanical stress and whether low-energy ESWs induced the nuclear translocation and activation of YAP1.

## 2. Materials and Methods

### 2.1. Participants

The study included 30 patients at the Senior Residence OPREPA Centre in Wrocław, Poland and was conducted from February 2017 to from March 2022. This was a prospective, interventional pilot study on the clinical evaluation and in vitro analysis of PUs. Approval from the Bioethics Committee of the Wrocław Medical University was obtained (approval no.: KB–632/2016 and approval date: 29 December 2016). All patients gave informed consent to participate in the study, which was carried out according to the Declaration of Helsinki and Good Clinical Practice guidelines. The study project was prospectively registered with the trial acronym SHOWN (SHOck Waves in wouNds) at the Clinical Trials Registry Platform (no. ACTRN12617000075381).

### 2.2. Qualification Procedure

The inclusion criteria were (1) a diagnosed wound with a PU etiology, (2) PUs with an area of more than 2 cm^2^ (allowing for the collection of biological material by biopsy), (3) PUs occurring for at least three months (qualified as chronic wounds), and (4) PUs classified as EPUAP grade II (partial-thickness damage of the skin) or EPUAP grade III (full-thickness damage of the skin and subcutaneous tissue damage with minor necrosis). The exclusion criteria included (1) chronic wounds of a different etiology than PUs (venous ulcers, diabetic feet, arterial or mixed ulcers), (2) PUs of areas less than 2 cm^2^, (3) PUs less than three months old, (4) EPUAP grade I (fading skin redness) or EPUAP grade IV (advanced muscle and bone necrosis), (5) PUs requiring urgent surgical intervention, (6) refusal to participate in the study, and (7) failure to comply with the study protocol.

### 2.3. Sample Characteristics

The group of 10 patients (9 women and 1 man) had a mean age of 85.8 years, a mean height of 170.2 cm, a mean body weight of 68.7 kg, and a mean BMI of 24.8 kg/m^2^. Most patients had significant physical limitations, senile dementia, cognitive disorders, and were self-care-dependent. Typical comorbidities were atherosclerosis (n = 6), arterial hypertension (n = 5), type 2 diabetes (n = 2), and venous insufficiency (n = 2). Three patients suffered from paresis after stroke (n = 3).

### 2.4. The Wounds’ Characteristics

All patients had diagnosed PUs, classified between grades II and III according to EPUAP, with a mean duration of 7.5 months. The anatomical location included the following areas: the sacral (n = 5), calcaneal (n = 3), and the trochanteric (n = 2). Planimetric assessment of wounds with a smartphone application (Swift Skin and Wound App., Swift Medical, Toronto, ON, Canada) at baseline showed a mean WSA of 13.5 (SD = 13.6) cm^2^, a mean length of 5.4 (SD = 3.0) cm, and a mean width of 3.5 (SD = 2.0) cm. Moreover, the clinical assessment using WBS showed a mean baseline score of 2.7 (SD = 2.4) points per 16 points, which was the maximum one could obtain (the higher score, the better clinical wound condition).

### 2.5. Therapeutic Intervention

All patients received two sessions of low-energy radial ESW (Cellactor^®^ SC1, Storz Medical, AG, Tägerwilen, Switzerland). The ESWs during sessions used the following parameters: a number of pulses of 300 at baseline + 100 per each cm^2^ of the wound surface area, a level of pressure of 2.5 bars, and an energy flux density (EFD) of 0.15 mJ/mm^2^, and a frequency of 5 Hz. The treatment protocol in our study consisted of two sessions of radial ESW administered twice a week, with a 3-day interval between sessions. Specifically, we assessed the wound tissue at three distinct time points: before the initiation of ESW treatment (M0), 24 h following the first session (M1), and 24 h after the second session (M2). The diameter of the ESW ballistic applicator was 15 mm. A sterile ultrasound gel was used as a coupling medium to reduce tissue resistance and to maintain adequate propagation of shock wave energy into the tissues. The gel was applied to both the wound surface and a sterile plastic barrier (sterile polyurethane film). The method employed during ESW involves utilizing the contact and labile technique with the applicator head. The initiation of ESW commences at the wound edges, progressing systematically towards the center, ensuring thorough coverage of the entire wound surface, including its base. All patients continued with standard wound care procedures using specialized materials, fluids, dressing agents, pressure relief mattresses and positioning.

### 2.6. Histomorphological Analysis

Tissue specimens were surgically collected from the wound area at a distance of 0.2–0.5 mm from the wound edge. The biopsy specimens were collected at baseline (M0) and 24 h after the first ESW (M1) and 24 h after the second ESW intervention (M2). The microscopic evaluation of the tissue before (M0) and after the ESW (M1 and M2) was performed on paraffin slides stained with H&E.

### 2.7. Immunohistochemical Assessment

The tested antigens (α-SMA, YAP1, Ki-67, and CD31) were visualized on tissue sections taken from the wound edge using standard immunohistochemical methods. Tissue sections were dewaxed and rehydrated. Recovery of antigens was accomplished by heating tissue sections in a microwave oven. Endogenous peroxidase was blocked by incubation in 3% H_2_O_2_. To prevent non-specific reactions, sections were incubated with 10% serum. Incubations with primary antibodies were carried out in a humid chamber at room temperature. Negative control was performed by omitting the primary antibody and incubating it with the serum. The antigen–antibody complex was visualized by applying a system Dako EnVision + HRP-labeled polymer anti-mouse system (Dako North America Inc., Carpinteria, CA, USA). The slides were contrasted in Mayer hematoxylin.

### 2.8. Immunohistochemical Evaluation

Stained preparations for the presence of YAP1 antigens and α-SMA were assessed given their staining intensity and percentages of cells with positive staining, according to Tuxhorn et al. [15]. For each tested specimen, the product of the percentage points and the rating of the intensity of the reaction was the “staining index”. This index was classified as zero (0), low (1–2), moderate (3–5), and high (6–9). The expression of the Ki-67 antigen was used to assess the proliferative status of the examined tissues, both in the epidermis and dermis. Under a 40X objective magnification in selected areas of tissues (0.1735 mm^2^), the number of nuclei cells that expressed the Ki-67 antigen was counted. The counts were performed in 10 areas with the maximum stain. The result was the number of cells with intensely stained nuclei. To assess the microvascular density, the immunocytochemical reaction for the presence of the CD 31 antigen (PECAM-1) was evaluated. The most intensely vascularized areas of the wound tissue (hot spots) were selected under the microscope magnification (×40). The number of stained vessels in representative surfaces was counted under high magnification (×400, 0.1735 mm^2^) on two different surfaces. Under high magnification, the fields of observation were subjected to cytometric analysis according to the following principles: Single immunoreactive endothelial cells or groups of other endothelial cells were counted as a single vessel. Endothelial staining in large intimal vessels and non-specific staining of non-endothelial structures were neglected in assessing wound micro-vascularization. The mean visual vascular density was calculated as the mean of the four measurements (two observers x two microscopic surfaces).

### 2.9. Statistical Analysis

Statistical analysis was performed using the GraphPad Prism 7.0 software package (GraphPad Software Inc., San Diego, CA, USA) and Statistica 13.3 (StatSoft Inc., Tulsa, OK, USA). Planimetric and clinical effects of the ESW intervention were verified by one-way repeated measures analysis of variance by ranks (the Friedman test). The effects of the ESW on microvascular density and proliferation index (Ki-67) were analyzed by one-way analysis of variance (ANOVA). When the one-way ANOVA results were significant, the differences between the evaluated parameters were examined with the post hoc test (Tukey’s multiple comparative test). The cross-tabulation along with the χ^2^ tests were used to compare the differences in immunocytochemical reactions to myofibroblast labeling (α-SMA) and YAP1 expression between three groups: the non-intervention group, the group with a single ESW intervention, and the group with two ESW interventions. *p* < 0.05 was adopted as a statistically significant level.

## 3. Results

### 3.1. Clinical Evaluation

**Figure 1:**
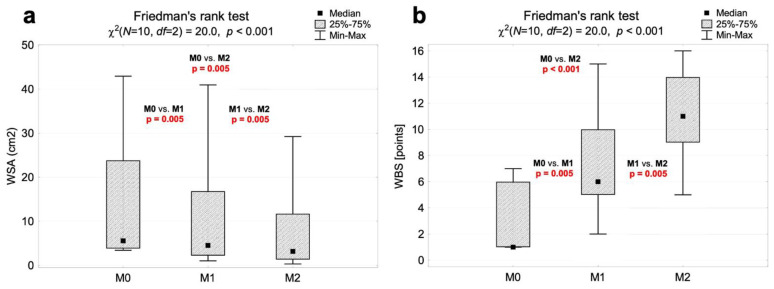
Results of planimetric and clinical evaluation. (**a**) WSA results before (M0) and after ESW intervention (M1, M2). (**b**) WBS results before (M0) and after ESW intervention (M1, M2). Abbreviations: WSA, wound surface area; WBS, wound med score, M0, measurement at baseline; M1, measurement after first ESW intervention; M2, measurement after second ESW intervention; M0–M1, comparison between M0 and M1; M0–M1, comparison between M0 and M2; M1–M2, comparison between M1 and M2.

The planimetric documentation for wound surface area (WSA) and clinical assessments using Wound Bed Scores (WBSs) showed significant improvements in wound-healing parameters. For the WSA assessment, there was a statistically significant (*p* < 0.001) reduction in the wound area by 3.6 cm^2^, 2.7 cm^2^ and 6.2 cm^2^ for the comparisons of M0 vs. M1 (*p* = 0.005), M1 vs. M2 (*p* = 0.005) and M0 vs. M2 (*p* = 0.005), respectively. For the WBS assessment, significant (*p* < 0.001) clinical improvements in the wound by 4.6 points, 4.0 points, and 8.6 points were noted, respectively, for the group comparisons of M0 vs. M1 (*p* = 0.005), M1 vs. M2 (*p* = 0.005) and M0 vs. M2 (*p* < 0.001). Detailed data are presented in Table 1 and Figure 1.

### 3.2. An Assessment of YAP Protein Expression 1

**Figure 2:**
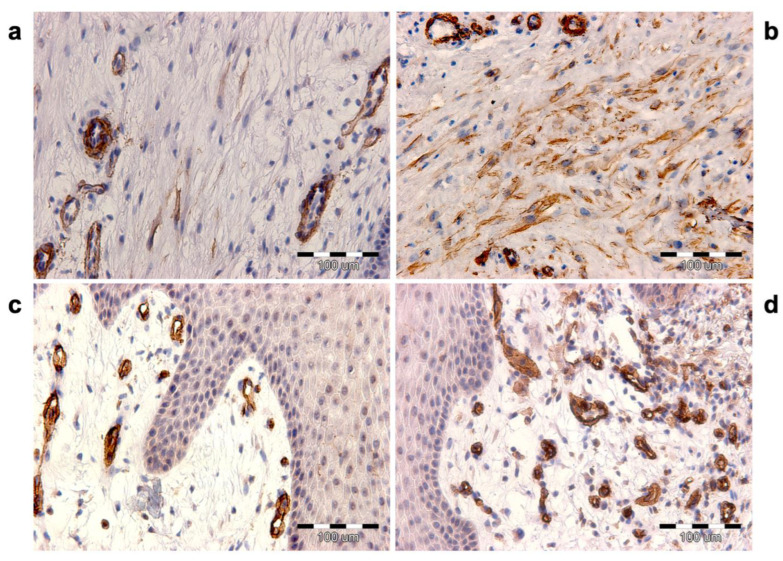
Immunocytochemical localization of alpha smooth muscle actin. (**a**) Before ESW (M0) intervention, only a few spindle cells are immuno-positive. (**b**) On the other hand, after ESW (M2) intervention, the number of cells showing a strong reaction increases significantly. The stained myofibroblasts are arranged in bands. A strong reaction is also present in the myocytes of the vascular wall. (**c**) Microvascular density; immunocytochemical localization of the CD31 antigen. (**d**) The number of vessels (CD31 positive) increases after ESW intervention.

**Figure 3:**
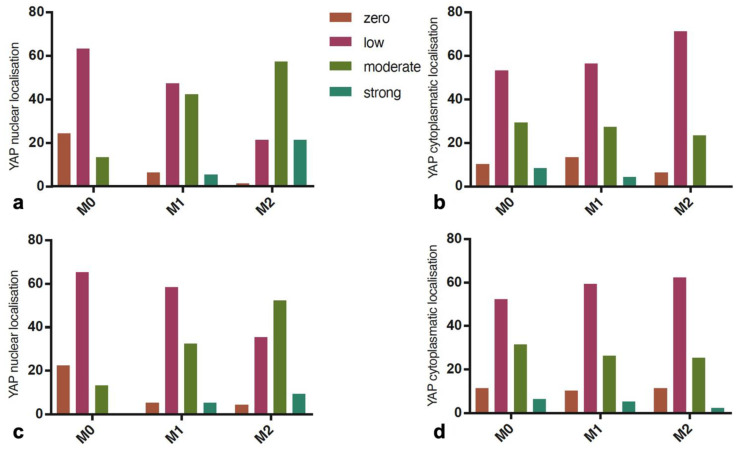
Distribution of YAP protein expression values. (**a**) Nuclear localization in the epidermis. (**b**) Cytoplasmic localization in the epidermis. (**c**) Nuclear localization in the dermis. (**d**) Cytoplasmic localization in the dermis. Abbreviations: YAP1, Yes-associated protein 1; M0, measurement at baseline; M1, measurement after first ESW intervention; M2, measurement after second ESW intervention.

An immunocytochemical evaluation of YAP1 protein expression was performed for ten patients with PUs (10 independent measurements for each tissue section). The presence of YAP1 was demonstrated in the nuclear and cytoplasmic localization both in the epidermis and dermis. For the area of the wound edge before ESW intervention (M0), the YAP1 protein was localized mainly in the basal layer of the epidermis in the nuclear and cytoplasmic localization (Figure 2a). The YAP1 antigen expression in keratinocyte nuclei before the ESW intervention (M0) was distributed with the following values: no expression, 24; low expression, 63; moderate expression, 13; and strong expression, 0 (Figure 3a). After the ESW intervention (M1), YAP expression in the nuclei of keratinocytes was visible both in the basal cell and lower layer of spinal cells (Figure 2b). The distribution of YAP1 expression values in keratinocyte nuclei was as follows: no expression, 6; low expression, 47; moderate, 42; and strong, 5. After the second ESW intervention (M2), YAP expression in keratinocyte nuclei was as follows: no expression, 1; weak expression, 21; moderate expression, 57; and strong, 21 (Figure 3b). Before the ESW intervention (M0), the values of YAP expression in the cytoplasm of keratinocytes were distributed as follows: none, 10; weak, 53; moderate, 29; and strong, 8 (Figure 3c). After the first ESW intervention (M1), the distribution of YAP protein expression values was as follows: no response in cytoplasm, 13; weak response, 56; moderate one, 27; and strong one, 4. After the second ESW intervention (M2), the values of the YAP protein expression were distributed in the following manner: no reaction, 6; weak reaction, 71; moderate reaction, 23; and strong reaction, 0. The distribution of YAP protein expression values at nuclear and cytoplasmic locations in keratinocytes is presented in Figure 2c. The analysis showed that the differences in YAP expression in keratinocyte nuclei (χ^2^ = 103.5, *p* < 0.0001) and the cytoplasm (χ^2^ = 14.36, *p* = 0.0259) were significant. YAP1 protein in dermis cells was localized in fibroblasts/myofibroblasts and vascular endothelial cells. The immunocytochemical evaluation revealed the antigen in both the cytoplasm and nucleus (Figure 2). Before the intervention of ESW (M0), the reaction intensity in the cell nuclei was distributed with the following values: no reaction, 22; weak reaction, 65; moderate reaction, 13; and strong reaction, 0. However, after the first ESW intervention (M1), the subsequent distribution of the reactions was obtained: no reaction, 5; weak reaction, 58; moderate reaction, 32; and strong reaction, 5. After the second ESW intervention (M2), the distribution of the reaction values was as follows: no response, 4; weak response, 35; moderate response, 52; and strong response 9. The distribution of values shown in Figure 3c was statistically significant (χ^2^ = 61.4, *p* < 0.0001). Before the ESW intervention (M0), the cytoplasmic responses to the YAP antigen in the dermis were distributed as follows: no reaction, 11; weak reaction, 52; moderate reaction, 31; and strong reaction 6. After the first intervention of ESW (M1), the distribution of reactions was as follows: no reaction, 10; poor reaction, 59; moderate reaction, 26; and strong reaction, 5. After the second ESW intervention (M2), the distribution of the intensity of immunocytochemical reactions to the YAP1 antigen was distributed with the following values: no reaction, 11; weak reaction, 62; moderate reaction, 25; and strong reaction, 2 (Figure 3d). The analysis of the cross-tabulation with the χ^2^ test indicated no significant results (χ^2^ = 3.732, *p* = 0.7129) (Table 2).

### 3.3. Myofibroblasts’ Activation

**Figure 4:**
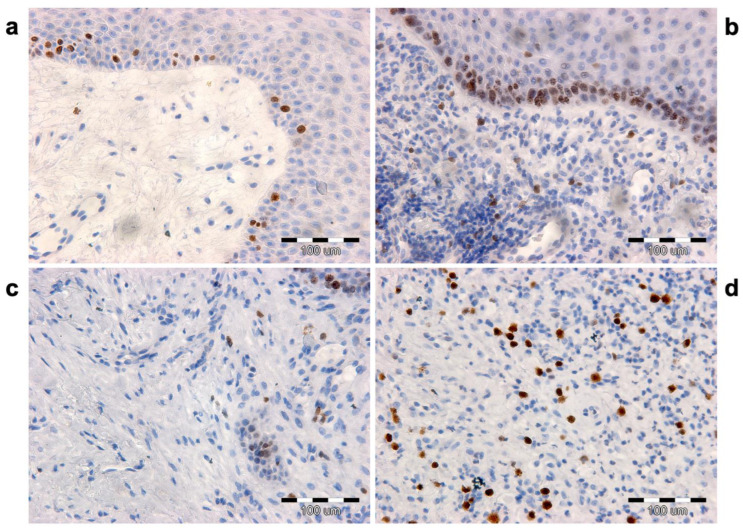
Proliferation index by detection of the Ki-67 antigen. (**a**) Before ESW (M0) intervention, only single basal layer keratinocytes are immuno-positive. (**b**) After ESW (M2) intervention, the number of proliferating keratinocytes in the basal layer increases significantly, and few cells are visible in the spinous layer. (**c**) Localization of Ki-67 antigen in skin connective tissue prior to ESW intervention (M0). (**d**) After ESW (M2) intervention, there is a significant increase in proliferating cells.

**Figure 5:**
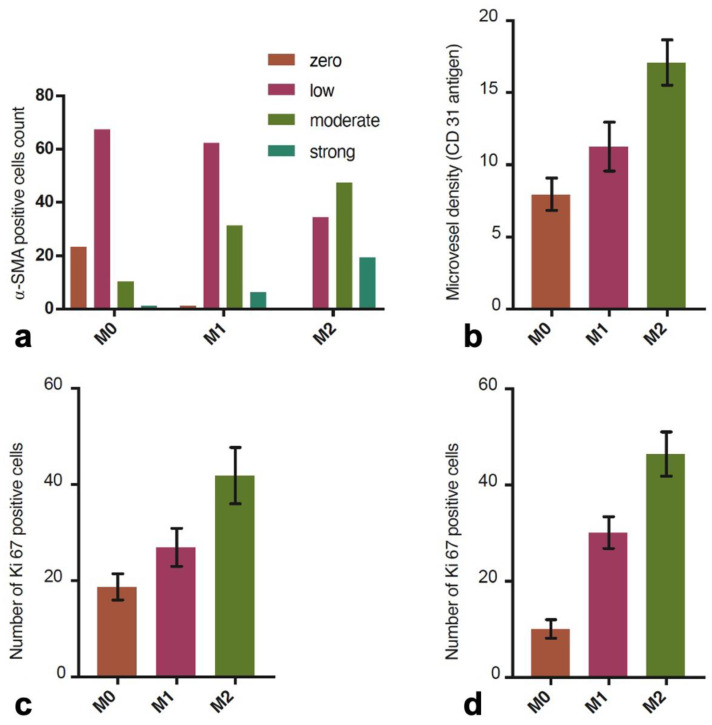
Distribution of alpha smooth muscle actin, micro-vessel density (CD31), and Ki-67 (proliferation index). (**a**) Distribution of smooth muscles’ alpha actin expression values (α-SMA) in miofibroblasts before and after ESW intervention. (**b**) Microvascular density before (M0) and after ESW intervention (M1, M2). (**c**) Proliferation index (Ki-67 antigen) before and after ESW intervention in the epidermis. (**d**) Proliferation index (Ki-67 antigen) before and after ESW intervention in the papillary layer of the dermis. Abbreviations: α-SMA, smooth muscle alpha-actin; CD31, cluster of differentiation 31; Ki-67, proliferation marker; M0, measurement at baseline; M1, measurement after first ESW intervention; M2, measurement after second ESW intervention.

Myofibroblasts were identified in dermis sections at the wound border by assessing the α-SMA expression in connective tissue cells and with the exclusion of blood vessel myocytes. Immunocytochemically visualized myofibroblasts arranged in bands were present in the wound bed (Figure 4). The results presented in the contingency tables are as follows. Before ESW (M0) intervention, there were the following results: no reaction, 23; weak reaction, 67; moderate response, 10; and strong response, 1. After the first intervention of ESW (M1), we found the following results: no response, 1; poor response, 62; moderate, 31; and strong response, 6. After the second ESW intervention (M2), the following results were obtained: no reaction, 0; weak reaction, 34; moderate reaction, 47; and strong reaction, 19. The results are shown in Figure 5. The contingency table analysis with the χ^2^ test showed significant results (χ^2^ = 97.11, *p* < 0.0001) (Table 2).

### 3.4. Proliferation Index

**Figure 6:**
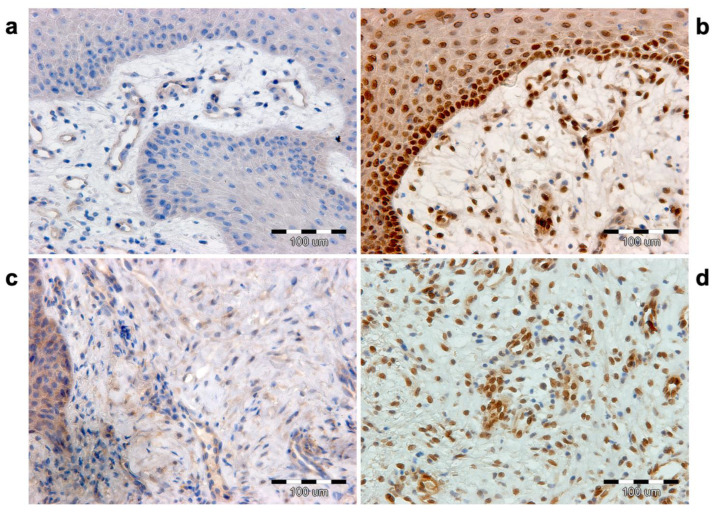
Immunocytochemical localization of the YAP protein. (**a**) Photo of a fragment of the skin before ESW intervention (M0); There is no reaction in the keratinocytes, while the reaction with a cytoplasmic localization is visible in the endothelium of the blood vessels of the papillary layer of the dermis. (**b**) YAP protein expression after ESW (M2) intervention is evident in the nuclear localization in the basal layer and in the spinous layer cells. (**c**) Localization of the YAP protein in the dermis before the intervention (M0). There is a weak reaction in the cytoplasm of fibroblasts and endothelial cells of blood vessels. (**d**) YAP protein localization after shock wave intervention. A strong reaction is localized in the cell nuclei of fibroblasts and in the nuclei of vascular endothelial cells.

Positive cells for the proliferative marker Ki-67 in the epidermis and dermis were counted on skin sections at the margin of the healing wound in three conditions: before ESW intervention (M0), after the first (M1) and after the second ESW interventions (M2) (Figure 6). Before the intervention, the mean number of positive cells in the epidermis was 18.74 (SD = 2.70, min = 12, max = 27) (Figure 3c). After the first and second interventions, there was an increased number of positive cells for the Ki-67 antigen for M1 (mean = 26.95; SD = 3.94, min = 19, max = 36) and for M2 (mean = 41.86, 5.84 min = 32, max = 57), respectively. The ANOVA test showed significant results (*p* < 0001). The mean numbers of Ki-67 positive cells in the examined dermis areas were, respectively, 10.08 (SD = 1.91, max = 15, min = 6) before the ESW intervention (M0), 30.07 (SD = 3.28, min = 21, max = 36) after the first intervention (M1), and 46.43 (SD = 4.57, max = 54, min = 38) after the second ESTW intervention (M2) (Figure 3d). The ANOVA test showed that these differences were significant (*p* < 0.001) (Table 3).

### 3.5. The Assessment of Microvascular Density

The assessment of microvascular density counted CD31 immuno-positive cells in the solid surfaces of the papillary layer of the dermis (Figure 4c,d). Before the ESW intervention (M0), the mean number of vessels at the wound edge was 7.96 (SD = 1.12, max = 10, min = 5). After the first ESW intervention (M1), the mean number of vessels increased to 11.26 (SD = 1.69, max = 15, min = 8). After the second ESW intervention (M2), the mean number of vessels increased to 17.09 (SD = 1.56, max = 21, min = 14) (Figure 5b). The obtained differences were significant, as indicated by the ANOVA (*p* < 0.0001) (Table 3).

## 4. Discussion

Cell proliferation index studies use the Ki-67 monoclonal antibody. The antibody stains nuclei in the cell cycle’s G1, G2, S, and M phases, while resting cells in G0, and cells entering the proliferation cycle remain unstained [16]. The monoclonal antibody Ki-67 (MIB 1) helps estimate the proliferative index of human tumors and provides essential information about the prognosis of neoplastic diseases [17].

Cell proliferation can be observed in cancer and physiological conditions such as wound healing. Applying antibodies for labeling the nuclear antigen in proliferating cells can assess skin-wound healing time. The advantage of the immunocytochemical studies of the Ki-67 proliferative antigen is to have parallel assessments of epidermal re-epithelization and changes in the dermis’s wound area.

Progenitor cells cannot always survive in a deep wound and participate in the regeneration of extensive wounds. Based on murine wound models, the abilities to produce large flaps of keratinocytes and re-epithelialize large wound surfaces are explained by epidermal proliferative unit (EPU) formation. The injury was observed to lead to the recruitment of IFE stem cells whose clones migrate from the periphery to the center of the wound surface [18].

Our findings on extensive PUs provide confirmation of keratinocyte proliferation in the epidermis. This confirms that re-epithelialization mainly depends on the migration of keratinocytes, which begins from areas of intact skin with increased proliferative activity [19]. In most cases, there was an increased number of proliferating basal cells for the regions of the epidermis adjacent to the wound edge compared to sites remote from the wound edge. For this reason, cells expressing the Ki-67 antigen were at these sites counted. Hair follicles and accompanying sebaceous glands, like other stem cells supporting wound re-epithelialization, were absent in the studied PUs and their vicinities. In this case, only the group of epidermal stem cells can participate in healing the wound.

After the ESW intervention, an increase in the proliferative activity of epidermal cells was observed. Moreover, Ki-67 antigen-positive cells were observed in the basal and spinous layers. After the first ESW (M1), the number of immuno-positive cells to the Ki-67 antigen compared to the baseline value (M0) increased by 43.81%. At the same time, an increase in proliferating cells was shown by 55.3% concerning the M1 condition and 79.57% concerning the M0 condition.

Fibroblasts are the most numerous cells of connective tissue, responsible for the synthesis, secretion, and remodeling of the ECM [20]. In the dermis at the wound edge, the study showed a significant increase in fibroblastic cells that expressed the Ki-67 protein. The finding of a greater number of Ki-67-positive fibroblasts in the vicinity of the wound edge and their decreasing number as they moved further from the wound edge indicated that intense fibroblast proliferation might contribute to the development of granulation tissue.

As a result of ESWs on wound healing, an increase in Ki-67-positive fibroblastic cells was observed. After the first ESW (M1), the rise in the number of immuno-positive cells in the dermis relative to the M0 condition was 198.31%. The number of Ki-67-positive cells after the second ESW (M2) compared to those after the first ESW (M1) increased by 54.40%. At the same time, this increase was 361.07% compared to immuno-positive cells before the ESW (M0).

For some skin wounds, an intense increase in the population of proliferating fibroblastic cells has been reported over 1.5 days post-wound. Due to the large variability in the number of positively stained fibroblasts in the intact dermis, the authors arbitrarily concluded that a three-fold increase in the number of immuno-positive cells to the cell proliferation marker Ki-67 should be considered a positive therapeutic effect [21].

This study showed an over three-fold increase in the number of proliferating fibroblastic cells after two ESW interventions. This finding confirms that ESW on hard-to-heal wounds can stimulate the proliferative activity of fibroblasts.

It should be noted that the cells with the fusiform shape typical of fibroblastic cells were subjected to immunocytochemical evaluation. Nevertheless, in the dermis area at the edge of the wound, lymphocytic infiltrates and proliferating endothelial cells may also show positive reactions to the proliferative antigen, making it difficult to assess the number of studied cells accurately [22]. Therefore, the study evaluated the myofibroblasts of wounds and their microvasculature. Fibroblasts in granular tissue differentiate into myofibroblasts by organizing the contractile apparatus in a process dependent on the interaction between cells and the extracellular matrix (ECM), growth factors, and mechanosensory signals [23,24,25]. The primary sources of wounded myofibroblasts are local fibroblasts in the intact dermis and subcutaneous tissue [26]. The increase in myofibroblasts in mice wounds usually begins on day 3 and peaks on day 7 after wounding [27]. 

Immunocytochemical analysis showed that wound exposure to ESW increased the number of myofibroblasts at the wound edge, often visible as integrated cell bands. The present results clearly show that the α-SMA expression intensity, which is a marker of myofibroblasts, increases progressively in response to ESW. The staining index for testing materials showed moderate and strong expressions of α-SMA; in other words, before the intervention, it was 11, after the first ESW it was 37, and after the second it was 66. These results correlated with the increase in proliferative activity after the ESW of connective tissue fibroblastic cells at the wound edge.

The microvascular density assessment was performed with the immunocytochemical detection of the CD31 antigen (PECAM-1). In the wound-healing process, PECAM-1 is involved in trans-endothelial migration, endothelial cell migration, and neoangiogenesis [28]. It has also been shown that mechanical stress forces strongly induce PECAM-1 activation. Histological studies showed a significant increase in PECAM-1 expression in endothelial cells in response to ESWs [29,30,31]. The present study showed increased vessels of the wounds after ESWs. After the first ESW (M1), the increase in the number of vessels was 41.46%, and after the second ESW (M2), it was 114.70%. This result indicates that ESWs can induce angiogenesis, resulting in better blood supply to the healing wound. The obtained results are consistent with in vitro studies, animal models, and clinical trials. Several studies report the improvement of wound vascularization and the reduction of ischemic necrosis due to ESWs. This effect was due to an increase in nitric oxide synthase (NOS) expression [32] and the activation of mechanosensitive angiogenesis pathways [11]. 

The present results indicate that ESWs increase the expression of the nuclear YAP protein both in the keratinocytes of the epidermis’s germinal layer, fibroblasts, and vascular endothelium. In both mouse and human epidermises, there is clear evidence for the association between the nuclear localization of YAP and the proliferative activity of epidermal stem cells [33,34,35]. Mouse wound models suggested an increased number of cells with nuclear YAP expression in the basal layer and the suprabasal cells of epidermal cells at the wound edge. In vitro studies of human keratinocytes identified YAP as an essential regulator of human keratinocyte proliferation [35]. There is an increased YAP expression at nuclear localization in skin wounds, while the knockout of YAP in the differentiated epidermis or YAP silencing in wounds slows their closure due to reduced proliferation [36]. Studies on mice wounds have shown that in the normal dermis, YAP is localized mainly in fibroblasts in the cytoplasmic localization, while in the wound bed, in the early healing phase, it takes on a nuclear localization [37]. In skin fibroblasts, YAP can respond to the sensing of the physical environment of the cell and influence cell proliferation, deposition, and the remodeling of ECM [38,39].

The major advance of the present research was that it studied human wounds. The results showed the increased expression of YAP in the nuclear localization after the ESW. Therefore, it points out the importance of mechanical forces in activating YAP. Furthermore, its translocation to the cell nucleus causes an increased proliferation of keratinocytes and fibroblasts, directly contributing to the intensification of chronic wound healing processes.

To sum up, in comparison with traditional wound-healing methods, ESW treatment offers unique advantages. The non-invasive nature of ESW therapy distinguishes it from surgical interventions, minimizing the risk of complications and promoting faster recovery. Moreover, the mechanical forces generated by ESW can stimulate the mechanosensitive pathways in skin cells, triggering enhanced cellular proliferation and extracellular matrix remodeling. Further research is needed to explore more precise molecular mechanisms, including changes in gene expression and the proteomics of signal transduction pathways. This mechanotransductive effect sets ESW treatment apart from conventional approaches, which may primarily focus on biochemical signaling. Additionally, the ability of ESW to penetrate deep tissue layers allows for a targeted and widespread impact on the wound bed. These distinctive characteristics position ESW as a promising modality in the pursuit of accelerated and effective wound healing.

### The Strengths and Limitations of the Study

The strengths of this study lie in its prospective design, enabling real-time data collection and analysis. A comprehensive approach was employed by combining clinical evaluation with the in vitro analysis of PUs, providing a thorough understanding of the subject matter. This multidimensional approach contributes to the advancement of translational medicine in wound management and underscores the significance of integrating diverse research techniques for a comprehensive understanding of the subject matter.

There are also some potential limitations to be discussed. The study included a limited number of 10 patients, which is a common limitation in pilot studies. This affects the generalizability of the findings. The research was conducted at the single center, which may impact the external validity of the results. Also, the study lacked a control group, and the sample size determination was based on patient availability rather than a formal power analysis, highlighting its exploratory nature. The unequal distribution of male and female participants (one man and nine women) could introduce gender-related biases.

To further advance the field of ESW therapy for PU healing, future research should focus on conducting larger-scale RCTs with diverse patient populations and control groups. Additionally, there is a need for comparative studies that compare ESWs with standard wound care interventions or other emerging therapies to establish its relative efficacy and cost-effectiveness. Investigating long-term outcomes, mechanisms at the molecular and cellular levels, and involving multicenter collaborations can contribute to a more comprehensive understanding of ESWs’ clinical impact and generalizability. Future studies should also incorporate patient-reported outcomes, assess the economic impact of integrating ESW therapy into routine wound care, and optimize treatment protocols based on iterative studies to maximize therapeutic benefits.

## 5. Conclusions

The ESW treatments in the examined wounds cause the activation of the mechanosensitive YAP transcription coactivator and its translocation to the cell nucleus and, as a result, an increase in the proliferative activity of epidermal cells, fibroblasts, and endothelial cells (Ki-67). Furthermore, the effect of ESW is also an increase in microvascular density (CD31) and the number of myofibroblasts (α-SMA). Consequently, ESWs promote the healing of PUs, in both aspects: the reduction of the quantitative parameters of the wound (planimetric assessment) and the improvement of the qualitative condition of the wound (clinimetric assessment).

## Figures and Tables

**Table 1:** Results of planimetric and clinical evaluation at individual measurement points after ESW intervention.

Outcomes	M0	M1	M2	*p*-Values
WSA [cm^2^]				<0.001
M ± SD	13.5 ± 13.6	9.9 ± 12.7	7.2 ± 9.1	
Me (Q1–Q3)	5.6 (3.8–23.8)	4.5 (2.2–16.8)	3.2 (1.3–11.7)	
Min–Max	3.4–42.9	1.0–40.9	0.3–29.2	
WBS [point]				<0.001
Mean ± SD	2.7 ± 2.5	7.3 ± 4.0	11.3 ± 3.6	
Median (Q1–Q3)	1 (1–6)	6 (5–10)	11 (9–14)	
Min–Max	1–7	2–15	5–16	

Abbreviations: WSA, wound surface area; WBS, wound med score, M, mean, SD, standard deviation; Me, median; Q1, lower quartile; Q3, upper quartile; Min, minimum; Max, maximum; M0, measurement at baseline; M1, measurement after first ESW intervention; M2, measurement after second ESW intervention.

**Table 2:** Distribution of wound YAP1 expression value and distribution of the immunocytochemical value of α-SMA evaluation in dermal cells after ESW intervention.

**Measurement**	**YAP1 Expression Level in Keratinocyte Nuclei of the Epidermis of the Wound Edge (n)**				**Together**	***p*-Value**
	Lack	Weak	Moderate	Strong		
M0	24	63	13	0	100	<0.0001
M1	6	47	42	5	100	
M2	1	21	57	21	100	
**Measurement**	**YAP1 Expression Level in the Keratinocyte Cytoplasm of the Epidermis of the Wound Edge (n)**				**Together**	***p*-Value**
	Lack	Weak	Moderate	Strong		
M0	10	53	29	8	100	0.0259
M1	13	56	27	4	100	
M2	6	71	23	0	100	
**Measurement**	**YAP1 Expression Rate in the Nuclei of Dermal Cells (n)**				**Together**	***p*-Value**
	Lack	Weak	Moderate	Strong		
M0	22	65	13	0	100	<0.0001
M1	5	58	32	5	100	
M2	4	35	52	9	100	
**Measurement**	**YAP1 Expression Level in the Cytoplasm of Dermal Cells (n)**				**Together**	***p*-Value**
	Lack	Weak	Moderate	Strong		
M0	11	52	31	6	100	0.7129
M1	10	59	26	5	100	
M2	11	62	25	2	100	
**Measurement**	**Values of α-SMA Assessment in Dermal Cells (n)**				**Together**	***p*-Value**
	Lack	Weak	Moderate	Strong		
M0	23	67	10	1	100	<0.0001
M1	1	62	31	6	100	
M2	0	34	47	19	100	

Legend: No reaction—0 points, weak reaction—1–2 points, moderate 3–5 points, strong 6–9 points. Abbreviations: YAP1, Yes-associated protein 1; α-SMA, smooth muscle alpha actin; M0, before ESW intervention, M1, after first ESW intervention, M2, after second ESW intervention.

**Table 3:** The number of positives for the Ki-67 antigen keratinocytes and the Ki-67 antigen of the dermis cells and the vascular density in the papillary layer of the dermis after ESW intervention.

Count of Ki-67-Positive Cells —Keratinocytes				*p*-Value		
	M0	M1	M2	M0–M1	M0–M2	M1–M2
M.	18.74	26.95	41.86	<0.0001	<0.0001	<0.0001
SD	2.70	3.94	5.0			
Min	12	19	32			
Max	27	36	57			
**Count of Ki-67-Positive Cells** **—Dermis Cells**				***p*-Value**		
	M0	M1	M2	M0–M1	M0–M2	M1–M2
M.	10.08	30.07	46.43	<0.0001	<0.0001	<0.0001
SD	1.91	3.28	4.57			
Min	6	21	38			
Max	15	36	54			
**Count of CD31-Positive Cells** **—Papillary Layer of the Dermis**				***p*-Value**		
	M0	M1	M2	M0–M1	M0–M2	M1–M2
M.	7.96	11.26	17.09	<0.0001	<0.0001	<0.0001
SD	1.12	1.69	1.56			
Min	5	8	14			
Max	10	15	21			

Legend: No reaction—0 points, weak reaction—1–2 points, moderate—3–5 points, strong—6–9 points. Abbreviations: CD31, cluster of differentiation 31; Ki-67, proliferation marker; M, mean, SD, standard deviation; Min, minimum; Max, maximum; M0, before ESW intervention, M1, after first ESW intervention, M2, after second ESW intervention; M0–M1, comparison between M0 and M1; M0–M1, comparison between M0 and M2; M1–M2, comparison between M1 and M2.

## Data Availability

The datasets generated during and/or analyzed during the current study are available from the corresponding author on reasonable request.
